# Increasing Seasonal Influenza Vaccination among University Students: A Systematic Review of Programs Using a Social Marketing Perspective

**DOI:** 10.3390/ijerph19127138

**Published:** 2022-06-10

**Authors:** Daisy Lee, Sharyn Rundle-Thiele, Tai Ming Wut, Gabriel Li

**Affiliations:** 1 School of Professional Education and Executive Development, The Hong Kong Polytechnic University, Hong Kong, China; edmund.wut@cpce-polyu.edu.hk (T.M.W.); tlgab.li@speed-polyu.edu.hk (G.L.); 2 Social Marketing @ Griffith, Griffith University, Nathan, QLD 4111, Australia; s.rundle-thiele@griffith.edu.au

**Keywords:** seasonal influenza vaccination, seasonal flu vaccine, flu immunization, university student, healthcare student, social marketing, systematic review, health behavior change, meta-analysis

## Abstract

The health and economic consequences of seasonal influenza present great costs to communities. Promoting voluntary uptake of the seasonal influenza vaccine among university students, particularly during the COVID-19 pandemic, can deliver protective effects for both individuals and the wider community. Vaccine uptake will be greatest when more of the social marketing benchmarks are applied. This systematic review summarizes evidence from programs aiming to increase seasonal influenza vaccination among university students. Six major electronic databases for health promotion studies (PubMed, EBSCO, ProQuest, Ovid, Web of Science, and ScienceDirect) were searched in November 2021 to capture peer-reviewed studies reporting field trials that have sought to increase seasonal influenza vaccination in university student populations, without any restrictions regarding the publication period. Following PRISMA guidelines, this paper identified 12 peer-reviewed studies that were conducted in the field in the United States, Australia, and Spain. Three studies were targeted at healthcare students and the rest focused on wider university student populations. Studies were narratively summarized, evidence of social marketing principles were identified, and quantitative outcomes were meta-analyzed. The findings indicate that none of the field studies, even a self-classified social marketing study, had adopted all eight of the social marketing benchmarks in program design and implementation. The two studies that only used promotion, but not other marketing-mix and social marketing principles, reported increases in students’ intention to be vaccinated but not actual behavior. Given that change is more likely when more social benchmarks are applied, this paper identifies activities that can be included in flu vaccine programs to improve flu vaccine uptake rates. The analysis highlights a lack of field studies focusing on increasing rates of vaccination behavior as research outcomes in countries beyond the United States.

## 1. Introduction

### 1.1. Significance of Increasing University Students’ Uptake of Seasonal Influenza Vaccine

Seasonal influenza (also known as seasonal flu) causes substantial morbidity and mortality in each winter influenza season. Prior to the outbreak of the COVID-19 pandemic, seasonal influenza killed about half a million people around the globe in 2018 [1]. Seasonal influenza is highly contagious and poses a well-documented risk. Severe influenza-like illnesses, hospitalization, and deaths occur mainly among high-risk population groups including young children, elderly people, pregnant women, patients with chronic diseases, and healthcare workers [1]. Hence, measures to prevent seasonal influenza and subsequent influenza-related illnesses and deaths among these high-risk population groups have received substantial focus from health promotion researchers, practitioners, and policymakers.

Although university students are a low-risk group for influenza complications and death, the highly contagious seasonal influenza still poses a severe health threat to university students and their close contacts. Seasonal influenza is a well-documented risk to university students [1], once infected, students suffer certain consequences that impact their well-being and people around them. First, students who are infected with influenza have a lower academic performance and their day-to-day social activities may be impacted [2]. Influenza-like symptoms, including sneezing, coughing, fatigue, and fever, can last for several weeks [1], affecting students’ class attendance, academic performance, and internship productivity [2]. Students suffering from influenza-like illnesses are unable to participate in social or extracurricular activities, which affects their social life and causes negative consequences on their wellbeing. Second, university settings are considered a hotbed for the spread of influenza and other viruses. Rates of spreading of seasonal influenza in universities can be high [3], given that hundreds of students can be packed in the same lecture theatre for hours, allowing a single sneeze or cough to spread the virus to everybody else attending the lecture. Due to the highly infectious nature of the virus, risks of spreading influenza on campus and in dormitories are recognized to be very high given the high proximity of students in class activities, club activities, social events, and a close living environment [4]. Moreover, an outbreak of seasonal influenza at a campus has the potential to spread into the community. Thus, preventing influenza infections among university student populations is beneficial to both students and the wider community in which they live [5].

According to the World Health Organization (WHO) [6], vaccination is the most effective measure to prevent seasonal influenza and subsequent influenza-related illnesses and deaths. Evidence indicates that influenza infections resulted from low seasonal influenza vaccination rates during the COVID-19 pandemic, further worsening the impact of the pandemic in terms of morbidity, mortality, and hospitalizations, causing significant healthcare and economic costs [7]. Thus, increasing annual seasonal influenza vaccination rates continues to be a top public health priority. Similarly, preventing the outbreak of seasonal influenza among university students through vaccination can minimize campus-wide influenza outbreaks.

Apart from the well-being of students, promoting seasonal influenza vaccination among university students could also deliver further protective effects for the community surrounding students. Whole communities can be protected from influenza viruses if a sufficiently high percentage of the population develop immunity to influenza [8]. Therefore, students who have immunity to influenza also play an important role in protecting their friends, family members, high-risk population groups, and other individuals in the community. Hence, increasing the vaccination rates among university students will help to increase overall coverage, thus contributing to the effort to achieve herd immunity [8] against seasonal influenza.

Although much research effort has been invested to understand the factors and barriers affecting university students’ attitudes and intentions to receive seasonal influenza vaccination [9,10,11,12,13], low seasonal influenza vaccination rates among university and college students are a global phenomenon [2,14,15]. A large attitude–behavior gap is observed. Only 30–40% of university students who claimed to have good intentions to get vaccinated eventually uptake the influenza vaccine [16,17]. Therefore, instead of surveying students on their reasons for receiving or not receiving the vaccination, effective programs that could promote vaccination behavior among university students need to be identified, and in time, replicated in the field across university settings. However, knowledge of vaccination programs that could effectively raise seasonal influenza vaccination rate among university students is limited. Filling this research gap, this systematic review’s first aim is to synthesize previous research attempts to unveil knowledge to understand how seasonal influenza vaccine uptake among university students has been encouraged.

### 1.2. Literature Assessment Method: Social Marketing Benchmark Criteria

Human behavior is complex and factors leading to low vaccine uptake are diverse. Healthcare researchers, practitioners, and policymakers have been investigating approaches to motivate influenza vaccination among people who have high vaccine acceptance and to promote vaccination uptake among those who hesitate. People who have high vaccine hesitancy usually “delay in acceptance or refusal of vaccination despite availability of vaccination services” [18], imposing a significant challenge to achieving herd immunity. While legislating compulsory vaccination can lead to public dispute and unrest, strategies that promote the voluntary uptake of vaccines may assist to encourage people who are hesitant about getting vaccinated.

Social marketing, among all approaches, has received a growing awareness among researchers for its effectiveness in addressing the issue of vaccine hesitancy [19,20]. The World Health Organization calls for application of social marketing to addressing low vaccination rates [21]. Social marketing, by applying commercial marketing principles, can be applied to effectively transform societies by enabling self-defined and self-determined actions in individuals that are aligned to the greater common interest of the society [22]. Social marketing differs from policy or public health approaches that impose mandatory measures (e.g., compulsory COVID-19 vaccination). Social marketing focuses on identifying what will move and motivate people to voluntarily take up a behavior. Unlike traditional expert-led approaches which typically focus on emphasizing health outcomes, social marketers understand the importance of creating “value” for people. User-centric approaches are applied to program design ensuring that calls to action are aligned with people’s interests [20].

Social marketing is an evidence-based approach that has been applied effectively to promote global health [23,24,25]. Social marketing interventions or programs comprising social marketing elements have been found to enhance voluntary vaccination behaviors including HPV vaccination in teenagers and college-age adults [26,27,28], COVID-19 vaccination [29,30], tick-borne encephalitis vaccination [31], and seasonal influenza vaccination among healthcare workers and young adults [15,31,32]. Despite the potential value of social marketing in influenza vaccination promotion, to date, there is no study aiming to synthesize evidence on the application of social marketing (or not) in field trials seeking to increase seasonal influenza vaccine uptake among university students. Therefore, this paper summarizes extant literature to fill this gap. Arguing that social marketing should be, in this case, university student-driven and leading to flu vaccine uptake among the target audience, Andreasen [33] first proposed six benchmarks with a key aim of differentiating social marketing from public health and other education efforts aimed at improving people’s health. From 2010, Andreasen’s six benchmarks were revised and extended, delivering eight benchmark criteria. The eight benchmarks proposed by the National Social Marketing Centre are behavioral change, segmentation, consumer-orientation, competition, theory, insight, exchange, and marketing mix [34].

The first benchmark criterion that a social marketing program has to fulfill is behavioral change, which requires that the intervention or program focus on delivering behavioral change within a fixed timeframe. The focus on behavior aims to ensure all efforts feature direct calls to action and extended program efforts beyond changing knowledge, attitude, or beliefs. The second benchmark, segmentation, distinguishes social marketing programs from other approaches. Social marketers who apply segmentation focus on identifying differences between people considering a range of demographic, geographic, psychological, or behavior characteristics [35] to ensure programs can effectively engage more people by catering to known differences. Segmentation exists in full when the study reports cluster analysis to identify segments with homogenous characteristics and design segment-specific interventions [36]. Third, consumer-orientation requires a social marketing program to be developed based on data and evidence. People and major stakeholders should be involved during the program planning process. A study demonstrates consumer orientation when it has applied any form of research to understand how people think, feel, and behave. Willmott et al. [37] proposed the MATE taxonomy to assess the extent to which people are involved in program design, development, and implementation. The MATE taxonomy categorizes the engagement of participants in program design along a low–medium–high continuum. The fourth criterion expects a social marketing program to consider what is in competition with the target behavior. Researchers should think about how existing programs (or the audience’s existing behavior) might provide similar benefits to the target audience, then find ways to deliver value above and beyond the identified competitors. Fifth, a social marketing program is expected to root in relevant behavioral theories to guide the development of the intervention. Insights, as the sixth criterion, would be derived from formative research and the above benchmarks to gain profound understanding of motivations, barriers, and enablers that could lead to the desired behavioral change. Insight exists when a clear understanding of what people are asking for emerges. Clear and actionable insights are usually devised from a higher level of consumer orientation [37]. A succinct summary of insights can serve as the guiding strategy for a program. The seventh criterion, exchange, acknowledges that two or more parties have to come together if behavior change is to be observed. Exchange is a core marketing concept and in a commercial setting products and services are sold at a price ensuring a firm makes a profit and a customer is satisfied after using the product. Social change is more complex given that benefits individuals may receive can be very distant (e.g., fewer deaths due to herd immunity) and exchange is most commonly thought of in terms of overcoming barriers and maximizing benefits. Lastly, the marketing mix principle encourages social marketers to extend beyond communication-only approaches. Recent evidence indicates that rates of behavior change are higher when a full marketing mix is applied [38]. The social marketing principle of marketing mix is most commonly understood as the 4Ps (i.e., product, price, place, and promotion) and many other frameworks exist to guide program planning.

Social marketing’s effectiveness as a behavior-change tool is widely established. Evidence indicates that behavior change is more likely when more of the social marketing benchmark principles are applied [39]. The second aim of this paper is to examine the extent to which core social marketing principles have been applied, with a view to further understanding how flu vaccination uptake may be maximized.

This systematic review aims to:

1. Synthesize knowledge gained from previous seasonal influenza vaccination promotion programs or interventions that have effectively increased university students’ vaccination rates;
2. Assess the extent to which social marketing principles were applied in identified studies;
3. Inform healthcare policymakers and university management on the effective practices that can be applied to motivate university students to uptake annual seasonal influenza vaccines.

## 2. Methods

This systematic review followed the guidelines stated in the preferred reporting items for systematic review and meta-analyses (PRISMA), outlined by Liberati et al. [40].

### 2.1. Search Strategy

This study searched for possible related studies in six major electronic databases: PubMed, EBSCO, ProQuest, Ovid, Web of Science, and ScienceDirect. The following key terms were used: ((seasonal flu vaccin* OR seasonal influenza vaccin*) AND (intervention* OR random* controlled trial OR evaluation OR trial OR program* OR behavio* change OR social marketing OR campaign* OR scheme* OR experiment* OR study OR studies OR assessment)). The search was performed initially in April 2021 before the development of a campus-wide influenza vaccination campaign and an updated search was completed in November 2021. No restriction was set on the publication type, published time and language during the initial search.

### 2.2. Inclusion and Exclusion Criteria

This review adopts the PICOS (population, interventions, comparator, outcomes, and study design) approach in accordance with PRISMA guideline [40]. The review question designed based on the structure of PICOS (Cochrane Handbook, Sec. 5.1.1) [41] is “for university students (P—population), how effective are seasonal influenza vaccination programs with more social marketing elements (I—intervention) as compared with programs with less benchmarks (C—compare) for improving students’ uptake of vaccine (O—outcome)?” To compare program design and effectiveness of prior interventions, this review did not limit the type of studies (S) to be included. Effectiveness of programs using randomized controlled trials (RCTs), case control study, or other experimental field study designs will be reported and analyzed with a meta-analysis.

In addition, the following exclusion criteria governed which studies were not eligible for this systematic review: (1) studies including target groups who are not college or university students; (2) studies with mixed results for college or university students and other target groups, with no data presented separately; (3) studies in which vaccination behavior is not measured as an intervention outcome; (4) studies with co-administration of other vaccines; (5) studies without a control group or baseline comparison; (6) studies evaluating pandemic influenza vaccines only; (7) studies not published in English; or (8) studies not published in a peer-reviewed journal, thus grey literature was excluded from this paper. Articles that meet any one of the exclusion criteria were excluded immediately with no further inspection.

### 2.3. Data Extraction and Synthesis

After the search was performed in the six databases to identify previous publications meeting the selection criteria, duplicated results were removed before the screening process. Two reviewers then worked independently through the screening process in full compliance with the inclusion and exclusion criteria stated in Section 2.2. After duplicated results were removed, 7347 unique articles were reviewed independently by the two reviewers based on whether the title and abstract of each article met the inclusion and exclusion criteria. When the two reviewers disagreed with each other, the full article was examined before final decisions were made.

**Figure 1:**
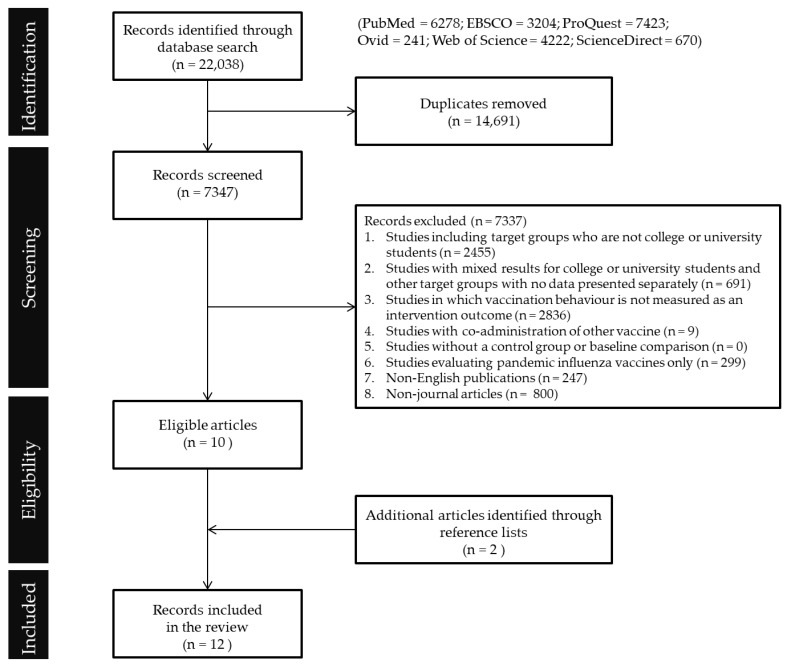
Flowchart of the literature search process (PRISMA).

Figure 1 shows the flowchart of the literature search process. A total of 2455 records were excluded since the studies were not targeting college or university students. A total of 691 records were excluded since university students were mixed with other population groups while the measured outcome among students could not be viewed separately from the whole population. A total of 2836 records were excluded for not measuring vaccination behavior as a program outcome. Nine studies were excluded because they involved co-administration of other vaccine(s). No studies were excluded due to the absence of a control group or baseline comparison against intervention group(s). A total of 299 studies were excluded since they were evaluating pandemic influenza vaccines only with no interventions concerning seasonal influenza vaccines. Finally, 247 pieces of non-English work and 800 non-journal articles extracted from the six databases were also excluded. After the initial screening process, the two reviewers inspected the full text of the 10 remaining articles to confirm their eligibility. Following full-text screening, a manual search was performed by reviewers to identity any additional manuscripts from the reference list of the extracted articles. Based on the above article inclusion and exclusion criteria, two additional articles were identified from the manual search. Including these two additional articles identified, 12 articles were included in this systematic review for analysis.

This review attempted to identify the presence of social marketing principles. This was achieved through examining reported study elements and cross-checking reported information against the eight social marketing benchmark criteria. A coding framework based on existing definitions of social marketing [34] were used to code the influenza vaccination programs in the 12 included studies for narrative synthesis. The research participants of each study were also identified to see whether the study targeted college or university students as a whole or whether the study targeted a specific group of students (e.g., healthcare students including medical, nursing, or physiotherapy students). Studies targeted at young adults at similar age groups as university students were also included to record prior learnings in influencing this age group.

## 3. Results

### 3.1. Study Characteristics

All the 12 studies reported programs targeting seasonal influenza vaccination behavior. Although we did not limit the search results using publication date, all the 12 studies were published in the last 10 years (i.e., from 2012 to 2021). Table 1 shows the location, duration, and target audience of the seasonal influenza vaccination program. Ten studies examined program effectiveness in increasing the uptake of seasonal influenza vaccine in the United States [16,42,43,44,45,46,47,48,49,50], one study was conducted in Australia [51], and one in Spain [52]. Results indicate that most studies were conducted in the USA and no studies from Asia, Africa, or South America were recorded. Of the 12 studies, 11 examined influenza vaccination program for one year [46]. One study evaluated a pilot program in the first year and interventions administered across a further two consecutive years. Of the studies examined, eight studies targeted general college or university student populations, three targeted only healthcare students such as nursing or medical students [42,51,52] and one targeted at young adults aged 18–27 [45].

All studies reported interventions or programs implemented that aimed to increase the vaccination rate of seasonal influenza vaccines among college or university students. The intended behavior outcomes were measured in terms of influenza vaccination rate (i.e., number of students vaccinated as a percentage of total number of students in the universities or schools). Table 2 shows the summary of seasonal influenza vaccination programs and behavior outcomes of the 12 studies analyzed. Nine studies recorded the size of student samples involved in the flu promotion program. In these nine studies, 27,655 students from 16 colleges or universities were exposed to the flu vaccination promotion programs or interventions [16,43,44,46,47,48,49,50,51]. Three studies did not report the total number of students involved in the flu vaccine promotion program or did not separate the student sample size from other samples in the study [42,45,48]. Three studies employed randomized controlled trial [16,45,49], seven studies used pretest–posttest study design [42,43,44,47,48,50,51], and two studies applied quasi-experimental studies [46,52].

### 3.2. Vaccination Program Assessment against Social Marketing Components

This review intends to synthesize knowledge from previous studies applied in the field to explore the effectiveness of seasonal influenza vaccination programs. Given behavior change rates are greater when more of social marketing’s eight core principles are applied, this review assessed the extent to which social marketing benchmarks were present in studies aiming to increase vaccination rates. Table 3 shows an assessment of the selected studies against eight social marketing components. None of the studies examined in this literature implemented all the social marketing components, which suggests there is ample opportunity to further extend study success by embedding social marketing principles in future studies.

Of the 12 included studies, only one study [48] was self-classified as social marketing. However, this study conducted by Hannings et al. only adopted the 4Ps marketing mix in the program [48]. Programs targeting healthcare students were found to use more social marketing components than those targeting university students in general. The three healthcare student programs [42,51,52] employed four to five social marketing components. However, for programs focused on all university students, four studies used only two (behavioral objective and 4Ps) [43,48,49,50], two studies used three components [44,45], one used four [47], and only two used five [16,46].

#### 3.2.1. Behavior Objectives and Outcomes

All nine studies [42,43,45,46,47,48,50,51,52] that implemented full-scale campus-wide or school-wide (e.g., program for the nursing school in a university) flu vaccination promotion programs reported an increase in flu vaccination rates compared to baseline (i.e., previous year(s) data) or control groups. Among the three studies that reported no significant change in vaccination rate, all studies used only a single communication channel to promote uptake of flu vaccine. In these single marketing communication channel studies, Roberto et al. [44] explored the impact of message appeal and Bronchetti et al. [16] tested variations of email message content among a group of recruited student samples. In both studies, student samples were exposed to the message once in the experimental conditions. Osborne et al. [49] examined the effect of flu vaccination content creation, exposure, and engagement in social media (Twitter) but found no significant difference in vaccination rate between student samples and the control group.

**Figure 2:**
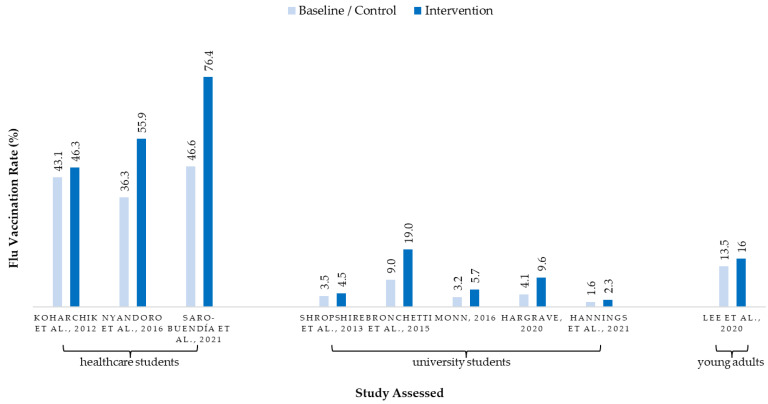
Seasonal flu vaccination rate (%) of included studies—intervention vs. baseline or control [16,42,43,45,47,48,50,51,52]. Remarks: Studies of Huang et al. [46], Roberto et al. [44], and Osborne et al. [49] are not shown on this chart as absolute vaccination rate was not provided in the literature.

Although full-scale campus-wide or school-wide promotion programs were found to effectively increase flu vaccination rates among university students by as much as 131% [47], the absolute increase in vaccination rate and update numbers was small. Figure 2 charts the flu vaccination rate of baseline (or control) and intervention results of studies according to the target audience types (i.e., healthcare students, university students, and young adults at the age of university students). Although all selected studies reported the percentage changes in flu vaccination rate against baseline or control, three studies [44,46,49] did not report the absolute vaccination rate. Among the studies that have reported absolute vaccination rate, four studies targeted at university students recorded a 0.3% to 5.5% increase against a small baseline (from 3.5% to 4.5% [43]; 3.2% to 5.7% [50]; 4.1% to 9.6% [47]; 1.6% to 2.3% [48]), showing that effective programs can boost the numbers of additional students to uptake flu vaccine. In Bronchetti et al.’s [16] study that randomized students into groups which received different email message content, the vaccination rate of the effective intervention group was significantly higher (19%) when compared to the control group (9%). However, unlike other studies that reported the vaccination rate of all students in the university, the double-digit vaccination rate only represented the outcomes among around 450 students who completed the post-campaign survey. For the three studies targeted at healthcare students with a larger baseline of vaccination rate, the program effectiveness was small (from 43.1% to 46.3%, +7%) [42] unless drastic measures were implemented (e.g., from 36.3% to 55.9%, +54% for programs run in both campus and placement sites [51]; from 46.6% to 76.4%, +64% for putting up identification of vaccination on staff ID at placement sites [52]).

**Figure 3:**
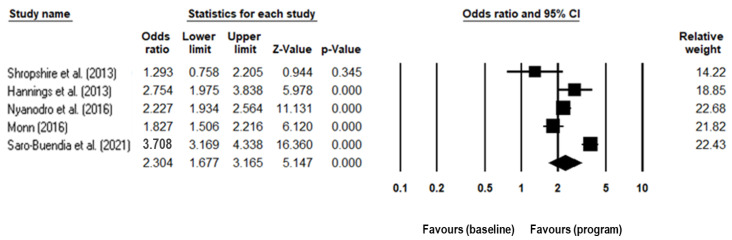
Forest plot of the meta-analysis showing the impact of vaccination interventions [43,48,50,51,52].

Statistical analyses of the impact of vaccination promotion programs on the vaccination rates reported in the included studies was conducted using Comprehensive Meta-Analysis version 3 [53]. Seven studies were not included in the meta-analysis because sample sizes or absolute vaccination rate are unavailable. Five studies [43,48,50,51,52] were included in the meta-analysis and the effect size representing the impact of the vaccination program is shown in Figure 3. The lower limit and upper limit indicate the 95% confidence interval. The size of squares in the forest plot of Figure 3 reflects the weight of the study according to the sample size. An odds ratio of 1.0 means that the vaccination rate was unchanged. While an odds ratio larger than 1.0 means that the vaccination rate was higher in the promotion group. The treatment effect was consistent across all studies included in the meta-analysis. All studies included in the meta-analysis had statistically significant effects in improving influenza vaccination behavior except for the study performed by Shropshire et al. [43]. The confidence interval of Shropshire et al.’s [43] study is wider than the others, reflecting that this study has lower precision. The *p*-value of Shropshire et al.’s study is 0.345 (*p* > 0.05) and does include the null value, indicating that the outcome of the study is not statistically significant. The effect was strongest (most distant from 1.0) in the program conducted by Saro-Buendía et al. [52] which used four out of eight social marketing benchmarks and a full 4Ps marketing mix in program design (OR = 3.71; CI: 3.17–4.34). Nyanodro et al.’s study [51] used five social marketing benchmarks and the full marketing mix (OR = 2.28; CI: 1.93–2.56). Hannings et al. [48] (OR = 2.75; CI: 1.98–3.84) and Monn [50] (OR = 1.83; CI: 1.51–2.22) also employed a full marketing mix in program design and significantly increased uptake of influenza vaccine. The pooled estimate represented by a diamond in Figure 3 shows that there is statistical significance at the meta-analysis level (Z = 5.147, *p* < 0.001). Influenza intervention program is better than control as the overall effect estimate, and its 95% confidence intervals are to the right of the line of no effect.

#### 3.2.2. Audience Segmentation

Segmentation categorizes audience population into groups based on similar demographic, geographical, psychographic, or behavioral characteristics [36]. Segmentation is an important initial step in program development as it allows practitioners to divide a heterogeneous population into small groups of audiences with similar characteristics and needs so that customized program elements can be offered to more effectively influence selected target group/segment(s) [54,55]. Since segmentation exists in full only when the study reports cluster analysis to identify segments with homogenous characteristics which are then used to design segment-specific interventions [36], none of the 12 studies assessed in this review conducted or referenced segmentation analysis. Three studies [42,51,52] reported a clear decision to target. These studies focused on healthcare students implementing programs targeted solely at nursing, medical, and healthcare students in campus and clinic placement sites. No consideration of differences within that student cohort were considered.

#### 3.2.3. Consumer Orientation

One or more target audiences should be ‘at the heart’ of a social marketing program and they should be actively involved in the program design and implementation to facilitate engagement, which is needed for behavior change to occur [56]. According to the MATE taxonomy [37], participant engagement is categorized along a low–medium–high continuum based on three factors: (i) agency, (ii) input, and (iii) empowerment. Low levels of consumer orientation are evident when methods requiring a low level of user involvement are used in research activities informing program design (e.g., surveys and interviews). Medium levels of participant engagement are delivered through focus groups, stakeholder consultation, co-design, and usability testing. To achieve the highest level of consumer orientation, methods such as peer leadership, peer education, and citizen science are examples of approaches used to effectively engage people. Higher levels of consumer engagement in program design were only evidenced in two studies. Nyandoro et al. [51] implemented a “peer-led, student-centered” campaign by involving student leaders or role models in the planning, visual design, and implementation of a vaccination campaign for all first year students at the school of medicine, nursing and midwifery, and physiotherapy. The peer champions, being educated with policy, efficacy and risks of flu vaccination, and information on convenient vaccination locations promoted the campaign at weekly lectures and on cohort-specific social media sites. Huang et al. [46] recruited and trained undergraduate students as HealthPALs (health peer advisors and liaisons) to provide in-person health outreach to promote flu vaccination in dormitories. The outreach program was designed and delivered by HealthPALs to promote the benefits of vaccination, provide information of the nearest flu clinic, and answer questions in their own dormitories through both online (e.g., dormitory-wide email and social media sites) and offline community (e.g., dining hall). The student HealthPALs also designed dormitory-specific posters to be displayed in common spaces and hallways. Two studies used low levels of consumer engagement in program design. Koharchik et al. [42] and Saro-Buendía et al. [52] surveyed the target audience in the previous flu season to understand factors and barriers leading to vaccination acceptance or hesitancy. Both studies formulated influenza vaccination programs based on the survey results. However, all the other eight studies did not involve student understanding or engagement in the program planning or implementation. Program design in these cases was based only on findings of previous literature that explored factors and barriers influencing the attitude and intention to get vaccinated.

#### 3.2.4. Competition

Competition to behavior change faced by the target audiences are considered and social marketing campaigns should report efforts aimed at minimizing competition [25]. In this review, direct competition is considered as flu vaccination options that are available outside university campus or clinic placement site (teaching and learning sites for healthcare students). Indirect competition was defined as activities that students considered as substitutes for flu vaccination to protect themselves from getting a flu [57]. Only three studies considered direct competition in their program design. As students could get free off-campus vaccination using their insurance plan, Bronchetti et al. [16] collaborated with some campus clinics to cover students’ vaccination fees by charging their insurance or parents. Similarly, Hargrave [47] partnered with private clinics that could bill students’ private health insurance to offer free on-campus flu vaccination to students. In contrast, Nyandoro et al. [51] emphasized to healthcare students the convenience of getting free flu vaccines at placement sites, as compared to vaccination at a GP clinic or retail pharmacy at a cost.

#### 3.2.5. Theory

Health behavior is complex and Rundle-Thiele et al. [58] posit that theory could provide an organizing framework for theoretically underpinned program design. Given that theories can be applied to explain and predict behavior, the clear application of theory is expected to enhance intervention effectiveness. Four studies mentioned the use of theory in the development of program strategy. Bronchetti et al. [16] adopted nudge theory and reported the positive effect of monetary nudges (financial incentives) and found that non-monetary nudges (peer endorsement and a coughing audio) had no effect on vaccination behavior. Huang et al. [46], based their program on the community health worker model and theories of peer influence [59]. This theory framework underpinned efforts that trained students to serve as ‘community health workers’ to influence their dormitory communities using their understanding of the community’s health needs, beliefs, and culture, and close proximity to them for their hallmates. Roberto et al. [44] examined the effect of fear-appeal messages designed based on the extended parallel process model and found that threat-, self-efficacy-, and response-efficacy-framed messages only affected attitude and intention to vaccinate. No influence on vaccination behavior was reported in this experimental study. Hargrave [47] designed the vaccination program by synthesizing the reasons why students determine or hesitate to get vaccinated from previous literature using the theory of planned behavior (TPB) as a contextual framework.

#### 3.2.6. Insight

Actionable insights derived from formative research including surveys, literature reviews, pilot studies, and stakeholder interviews could inform effective program design to influence desired behavior [60]. Insight exists when a clear understanding of what people are asking for emerges. Only two studies [46,51] reported to have acquired new insights in the pre-planning process. Both studies reported a high level of participant engagement to inform program development. Huang et al. [46] sought to gain knowledge from a pilot study in the previous flu season to enhance program effectiveness in increasing vaccination rate. Two studies [42,52] conducted a cross-sectional survey in the previous influenza season to understand the reasons for receiving and not receiving seasonal flu vaccine among their target audiences. Barriers including cost and convenience of vaccination were addressed in the planning and implementation of the program. The factors and barriers identified were identified using a cross-sectional survey and a more nuanced understanding could have been obtained using design methods. All remaining eight studies were not audience-oriented and no clear reporting of insights emerging from research were evident.

#### 3.2.7. Exchange

Social marketing also proposes offering tangible (e.g., incentive to get vaccinated) or intangible (e.g., recognition for healthcare students to be responsible for their patients) benefits as an exchange to motivate target segment(s) to engage voluntarily with the program [33]. Four studies explicitly employed tangible and intangible exchanges to motivate vaccination behavior. In Koharchik et al.‘s study [42], nursing students who got vaccinated would enter into a lucky draw for gift cards to the university bookstore. The post-campaign survey indicated that raffles were one of the reasons for the observed increase in vaccination rates. In Bronchetti et al.’s study [16], participants were randomly assigned into a control group and three intervention groups. Participants in one of the intervention groups could receive $30 cash if they received a flu vaccine at the campus health center by a specific date. Results indicated that the vaccination rate of the intervention group (19%) was significantly higher than the control group (9%) and the other intervention groups (8% and 9%) that received no exchange items for vaccination. Lee et al. [45] tested the effectiveness of a vaccination in-app reminder message (with or without loyalty points rewards for vaccination) through a healthcare provider mobile app. This study found that incentive in the form of in-app points had a similar effect as a no-incentive general reminder in increasing vaccination rates when compared to the control group (people who did not receive any vaccination reminder), providing further evidence on the effectiveness of financial incentives. Differing from the above three studies that used financial exchange, Saro-Buendía et al. [52] implemented a program to acknowledge medical students who were vaccinated by putting a sticker on their staff ID badges in their clinical placement hospital. This was regarded as a recognition for being responsible and willing to protect their patients from influenza.

#### 3.2.8. Marketing Mix (4Ps)

In a social marketing intervention program, the 4Ps of commercial marketing mix (product, place, price, and promotion) should be used whenever the approaches are financially feasible [38]. Table 4 summaries the use of marketing-mix implemented in the studies included in this review.

Product, in this systematic review, refers the advocated behavior of getting vaccinated [61]. Since all studies included in this review were selected based on their behavioral change objective to influence seasonal influenza vaccination, all the 12 studies provided target audiences with a tangible product (i.e., the flu vaccine).

Place refers to where the vaccine is administered and ideally ensuring that a convenient location is made available to support students to obtain vaccinations [61] (e.g., some successful vaccination campaigns for healthcare workers administered vaccines at high traffic locations such as the hospital canteen [62]). Except for the two studies [44,49] that did not report a significant behavioral change in vaccination rates for the intervention groups against control groups, all the other 10 studies made additional arrangements to make flu vaccination easily assessable for students. In the three studies targeted at healthcare students [42,51,52], flu vaccination could be received on campus or at clinical placement teaching areas, ensuring that obtaining vaccines was easy and convenient for students. Apart from an on-campus vaccination administered by flu clinics and health centers [16,43,50], additional clinics set up in dormitories [46] and mobile flu shot clinics [47,48] were implemented to facilitate vaccination. Lee et al. [45] reminded target audiences to get a flu shot at pharmacies that students would visit to pick up prescriptions.

Price involves all costs and benefits associated with seasonal influenza vaccination [61]. Previous research revealed that ‘willingness to pay’ for the vaccine is a major barrier to seasonal flu vaccination particularly among those who have high vaccine hesitancy or [63,64]. Apart from the two studies [24,29] that did not report a significant change in vaccination rate, all studies offered free vaccine to students either subsided by the university or billed to students’ private health insurance.

Promotion influencing vaccine acceptance includes audience-centric creative strategy (i.e., advertising message and visuals), communication channel strategy, and source strategy (i.e., spokesperson or influencer) [20]. The use of promotion channels and message strategy of the studies included in this review are summarized in Table 4.

Roberto et al.’s study [44] that exposed participants to different framing of fear-appeal advertisement was found to increase intentions to vaccinate but not subsequent vaccination behavior. Osborne et al. [49] engaged participants with vaccination promotion content in Twitter for eight months throughout the flu season but was also unable to increase vaccination uptake. Hargrave [47] did not reported how the vaccination program was promoted. All nine studies [16,42,43,45,46,48,50,51,52] that effectively increased vaccination rates used multiple audience touch points across online (including email, school and health center website, social media, and mobile app) and offline (including posters, education talks, leaflets, radio, university publications, and personal selling) marketing communication channels. These nine effective programs implemented different message strategies. All studies targeted at healthcare students [42,51,52] used moral appeals as a message strategy with taglines emphasizing student’s responsibility to be vaccinated to protect themselves and their patients. In the peer-led campaign intended to influence students living in the same dormitory, Huang et al. [46] promoted vaccination as a protection for students and fellow hallmates using community identity and sense of collective responsibility. Bronchetti et al. [16] and Lee et al. [45] persuaded audiences to receive vaccination through cash incentives and in-app loyalty points respectively. However, three studies [43,48,50] encouraged students to be vaccinated without featuring the benefits or costs of flu vaccination. These campaigns provided information on dates and locations of clinics.

## 4. Discussion

### 4.1. Lack of Studies on Vaccination Behavior in Countries beyond USA

Increasing seasonal influenza vaccination rates among university students is an important public health agenda for campus communities and broader society. Research reporting solutions that deliver evidence-based approaches that can be applied to increase vaccine uptake are lacking. According to the PRISMA flowchart (Figure 1), most extant literature on motivating seasonal influenza vaccination either focused on behavioral outcomes among high risk populations or factors affecting university students’ attitudes or intentions that usually do not turn into actual vaccination behavior [16,17]. Thus, although a vast number of studies have investigated how to increase the uptake of seasonal influenza vaccine, only 12 studies published to date report tested approaches that have been applied with the aim of increasing vaccine uptake. Moreover, 10 out of these 12 studies were conducted in the USA and only one was implemented in Australia and one in Spain, indicating a huge research gap to understand effective approaches that can be applied in countries other than the United States. Lack of theory-informed program design and the use of RCT.

Health behavior is complex. As theories could provide an organizing framework to explain and predict behavior, the clear application of theory is expected to enhance intervention effectiveness [58]. However, only four of the eligible studies mentioned the use of theory in program design and implementation. To enhance the effectiveness of vaccination programs, the use of health behavior theory is recommended for future studies.

Among all studies on vaccination behavior, there are only three RCTs in eligible studies. Thus, the internal validity is quite low in eligible studies and the effect size may be overestimated. Hence, more RCTs are required in future studies.

### 4.2. Lack of Audience-Centric Approach in Program Design

Rundle-Thiele et al. [56] showcased how the eight core social marketing benchmarks can be applied using the co-create-build-engage (CBE) process to effect voluntary health behavior change. In the pre-planning stage, programs should be co-created with people (e.g., college and university students) and other key stakeholders who are typically involved in influenza vaccination uptake. For on-campus programs intended to motivate seasonal influenza vaccine uptake, segmentation should be used to identify heterogenous groups among university students and competition to vaccination behavior should be assessed. Theoretically underpinned formative research should be conducted to derive student-oriented insights to guide program planning and implementation.

None of the studies assessed in this review have conducted or referenced segmentation analysis to identify homogeneous groups sharing similar characteristics in the target population. The three studies [42,51,52] focused on healthcare students only evidenced the use of targeting in which decisions were made to target students in researchers’ discipline (e.g., nursing school). Although healthcare and non-healthcare students have documented differences in the reasons behind influenza vaccine acceptance [13,65], interventions based on group difference within the student population were not reported. According to an advanced multivariate segmentation analysis conducted by Lee et al. [66], university students should be segmented. The Lee et al. [52] study identified four heterogenous groups with observed differences in receptivity of influenza information and vaccine acceptance. The Lee et al. [66] study identified that at least half of the students have strong vaccine hesitancy and vaccination behavior cannot be influenced simply by provision of education, peer-influence, or incentives. To date, no studies have assessed tailored intervention programs developed based on segment differences in vaccine acceptance or information receptivity. ‘One-size-fit-all’ programs were developed in all the studies reviewed in this paper.

Only three studies [16,47,51] considered direct competition from off-campus influenza vaccination that students could receive vaccines free of charge using their private insurance plan. None of the studies explored and incorporated indirect competition or perceived substitution for influenza prevention (e.g., healthiness [42]) in program design.

Although four studies mentioned the use of theory underpinning program development, only Bronchetti et al. [16] delineated how nudge theory was used to guide the design of their experimental conditions. Recent evidence demonstrates greater effect sizes when programs are built on theory [67]. Moreover, only three studies [42,51,52] targeted at healthcare students conducted surveys prior to program implementation to derive student-oriented insights for program development. Only Huang et al. [46] conducted a pilot study to inform and improve program design.

Accumulating evidence from social marketing indicates that programs should be designed with, and not for, target audiences [56]. Only two studies (Nyandoro et al. [51] and Huang et al. [46]) employed high levels of customer orientation. These studies designed a peer-led student-centered vaccination program together with student leaders. Two studies used low level participant engagement mechanisms (i.e., surveys) and all other eight studies were not consumer-oriented, indicating a lack audience-centric approach in influenza vaccination program planning. Future programs aiming to increase seasonal influenza vaccination rate are recommended to have medium to higher level of student participation in program design (e.g., co-creation or co-design, peer-leadership or peer-education).

### 4.3. Building Programs That Engage Students for Behavior Change

Elements of the marketing mix (4Ps: product, place, price, and promotion) should then be built based on insights from the co-create process to engage students in an exchange for voluntary vaccination behavior. Two studies (Osborne et al. [49] and Roberto et al. [44]) developed the research hypotheses without adopting any of the social marketing principles and built their vaccination programs only around theoretically hypothesized promotion tactics. Although participants’ attitude and intention to be vaccinated were reported to increase, their programs had no significant effect on actual vaccination behavior. All 10 other studies assessed in this review implemented comprehensive marketing mixes and recorded significant increases in influenza vaccination rates delivering further evidence that approaches adopting a full marketing mix are more effective than communication only approaches.

Table 5 summarizes key learnings from these studies that positively improve uptake of seasonal influenza vaccine. Since authors of the 12 included studies only reported the overall changes in vaccination rate, the key learnings about effective and ineffective program activities summarized in Table 5 are based on learnings concluded by the authors in the selected studies. The key learnings summarized are qualitative comments with no empirical substantiation from the selected studies.

Price (free or incentive that offset the vaccine fee) and place (convenient locations and hours) were found to be the most important components of an effective vaccination program. While monetary incentives deliver a strong nudge to vaccinate, non-monetary nudges have no effect. For promotion, students should be reached using multiple online and offline communication channels. While online channels such as a school website and email were found to be effective, the use of social media may not affect vaccination behavior. This finding echoes with the study of Benis et al. [68] which found that heavy social media users are exposed to negative influenza information over social media resulting in low vaccination rates. Although direct email or mobile messaging is useful in communicating vaccination programs, repeated messages are found to have diminished effect. In a study [45] where participants received multiple in-app messages throughout the program period, the majority of participants responded to the vaccination messages and were activated immediately after receiving the first message. This phenomenon matches with important findings from a segmentation study conducted by Lee et al. [66] that students with strong vaccine hesitancy will not respond to the same message no matter how times they are exposed to it. Direct repeats of the same message will only work for certain types of students. Notably, reminder messages were used to remind students who have indicated their intention to receive vaccination to take action. Reminder messages were found to effectively mobilize the segments who have high vaccine acceptance to act. Education, as proposed in many health promotion studies, was found to have no effect on increasing vaccination rates [20]. As per the segment characteristics identified in the study of Lee et al. [66], university students who have strong vaccine hesitancy will not be influenced by the provision of information and education and students who have strong vaccine acceptance do not need to be educated for action. Moreover, for those who are receptive to influenza vaccination, positive influence by parents, peer, and healthcare center staff would be influential to vaccine uptake.

## 5. Conclusions

This paper is the first systematic literature review to synthesize extant knowledge in peer-reviewed journals of social marketing application in seasonal influenza vaccination interventions targeting university students, using the eight social marketing benchmark criteria. Although only one study self-identified as using social marketing, most programs that effectively increase influenza vaccination rates were found to adopt a full marketing-mix and a few other social marketing components. A lack of behavior change studies in countries beyond the USA is highlighted. Future studies that fully exploit the potential of social marketing in program design and implementation would enable policymakers and university healthcare promotion personnel to improve influenza vaccine uptake on university and college campuses. In particular, omission of segmentation, students’ active involvement in program planning and/or delivery, consideration of competition, formative research for insight generation, provision of incentives for exchange, and the lack of theory-driven program design were highlighted as areas for improvement that may be capable of yielding even higher vaccine uptake rates.

Despite its contribution, this paper has several limitations. This review focused on university students and on-campus programs. Since students’ decision to receive influenza vaccine can also be influenced by off-campus factors from family members and friends, propaganda about vaccine in social media and other online platforms, community health support, personal health insurance, future research can explore a system-approach of program design to holistically influence vaccination behavior. For the meta-analysis, not all the selected studies were included because sample sizes were not provided in some studies. The treatment effects of intervention programs might vary across the studies although as different social marketing benchmarks and marketing mix were applied. In addition, those studies span across last decade. The mind-set of the people might change as time goes by. Lastly, this review did not include grey literature although relevant vaccination program learnings and evaluations can be found in the grey literature. Future research can consider synthesizing informal assessments reported in the grey literature to further benefit development of better influenza vaccination programs for students.

## Figures and Tables

**Table 1:** Characteristics of included studies.

Study	Location	Duration of Vaccination Promotion Program	Target Audience
Koharchik et al. (2012) [42]	USA	2011–12 flu season	Healthcare students
Nyandoro et al. (2016) [51]	Australia	2014 flu season (March–June 2014)	
Saro-Buendía et al. (2021) [52]	Spain	2018–19 flu season (October 2018–March 2019)	
Shropshire et al. (2013) [43]	USA	2011–12 flu season (September 2011–February 2012)	University students
Bronchetti et al. (2015) [16]	USA	2012–13 flu season (October–December 2012)	
Monn (2016) [50]	USA	2014–15 flu season (September–December 2014)	
Huang et al. (2018) [46]	USA	Pilot program: 2013–14 flu season	
		Enhanced program: 2014–15 and 2015–16 flu seasons	
Roberto et al. (2019) [44]	USA	NA	
Hargrave (2020) [47]	USA	2018–19 flu season (September 2018–February 2019)	
Osborne et al. (2021) [49]	USA	2018–19 flu season (October 2018–May 2019)	
Hannings et al. (2021) [48]	USA	2018–19 flu season	
Lee et al. (2020) [45]	USA	2016–17 flu season (September–November 2016)	Young adults aged 18–27

**Table 2:** Details of seasonal influenza vaccination programs and behavior outcomes.

				Behavior Outcomes (Vaccination Rate %)	
Study	Target Audience and Sample Size (n ^1^)	Study Design	Details of Seasonal Influenza Vaccination Promotion Program	Absolute	Difference vs. Baseline ^2^ and/or Control
Koharchik et al. [42]	Nursing students (*n* = unknown)	Pretest–posttest design	(1) Posters from the CDC urging immunization; (2) educational information shared in post-clinical conferences; (3) emails reminding dates and convenient locations of influenza clinics on campus; (4) a draw for gift cards to the university bookstore for vaccination	Intervention: 46.3% Previous year: 43.1%	+7%
Nyandoro et al. [51]	First-year medicine, nursing and midwifery, and physiotherapy students (*n* = 1620)	Pretest–posttest design	(1) Peer champions (who were trained with the evidence base behind the policy, efficacy and risks of influenza vaccination, information about convenient locations where students could get flu vaccination) delivered weekly reminders at lectures and through cohort specific social media outlets; (2) information pamphlet (printed and electronic) for peer champions and university staff to disseminate; (3) university staff, both on campus and at clinical placement sites, promote annual flu vaccination to the students.	Intervention: 55.9% Previous year: 36.3%	+54%
Saro-Buendía et al. [52]	Medical students of two universities (*n* = 1490)	Quasi experimental study	For both control and intervention groups: (1) educational materials (talks, posters, leaflets, and videos); (2) access to the vaccine (flexible hours, mobile teams, and free vaccination); (3) promotion of vaccination by HCWs; (4) rewards (badges and lanyards). Activities only for intervention group: (5) educational talks by senior years students; (6) flexible hours, mobile vaccine teams in teaching areas of the hospital; (7) reminder messages via Facebook and WhatsApp; (8) sticker on staff badge to identify those who have vaccinated.	Intervention: 76.4% Previous year: 46.6% Control: 55.3% Previous year: 42.7%	+27%
Shropshire et al. [43]	Undergraduate students (*n* = 720)	Pretest–posttest design	(1) Flyers, which had been implemented in previous year, were posted across campus (key locations such as dormitories, campus gym, and student union); (2) PowerPoint reminder slide (to be displayed by faculty members in class); (3) web banner on health center website, campus homepage, and campus Web portal and social media pages.	Intervention: 4.5% Previous year: 3.5%	+28%
Bronchetti et al. [16]	Students from six colleges (*n* = 9358)	Randomized controlled trial	Students were randomized into one of four conditions. Each group received the same number and timing of e-mails (one initial e-mail and two reminders) but the e-mail content was different. Group 1 (control)—on-campus vaccination information; Group 2 (incentive)—informing students that they could receive $30 cash if they get a flu vaccine at the campus health center by a specific date Group 3 (peer)—emails were sent from peer endorsers to recommend receiving flu vaccination Group 4 (coughing)—emails began with the text “Listen to this 3-s clip and imagine feeling like this during finals week!” with a link to an audio file of a sick person coughing.	Group 1 (control): 9% Group 2 (incentive): 19% Group 3 (peer): 8% Group 4 (coughing): 9%	No significant difference between group 3 (peer), group 4 (coughing), and the control. Group 2 (incentive): +119% * only +34% vs. control if cannibalization of off-campus vaccination is considered
Monn [50]	Post-graduate and undergraduate (*n* = 5248)	Pretest–posttest design	(1) Launched three vaccination clinics; (2) influenza information and reminders for immunization clinics were promoted on student health center Facebook page, college website, and campus-wide posters; (3) health center staff’s recommendation to students visiting the center.	Intervention: 5.7% Previous years: 3.2%	+78%
Huang et al. [46]	Students in 4 of the 6 dorms (*n* = unknown)	Quasi experimental study	Pilot: Health Peer Advisors and Liaisons (HealthPALs) greeted students at dining hall, informed them of the flu clinic, explained the benefits of vaccination, and answered students’ questions.	NA	+66%
	Undergraduate students (*n* = 6635)		Intervention: In addition to (1) in-person outreach, HealthPALs conducted (2) a personalized social media campaign. (3) Dormitory-specific posters encouraging fellow residents to attend the nearest flu clinic. (4) HealthPALs distributed outreach materials through dormitory-wide email lists, social media websites, and printed postings in common spaces and hallways. (5) a new flu clinic.	NA	+85% * +58% if effect of newly added clinic is excluded
Roberto et al. [44]	College students *n* = 482	Pretest–posttest design	Participants were randomly assigned to one of the four message exposure conditions: 2 (high threat/low threat) × 2 (high efficacy/low efficacy) Attitude and intentions towards flu shot were measured immediately after reading the message. Flu shot behavior was measured 30 days later.	HTHE: 20% HTLE: 17% LTHE: 23% LTLE: 16%	No effect for fear appeal messages (either efficacy or threat or their interaction effect) on behavior
Hargrave [47]	Undergraduate students (*n* = 1400)	Pretest–posttest design	(1) Implemented a one-day mobile flu shot clinic on campus; (2) partnered with local pharmacy that could bill students’ private health insurance (i.e., vaccination cost was reduced from USD20 to free of charge)	Intervention: 9.6% (no mobile clinic): *4.4%* Historical average: 4.1%	+131% vs. 6-year average * +7% (if increase from mobile clinic is excluded)
Osborne et al. [49]	Undergraduate students (*n* = 702)	Randomized controlled trial	Control group—participants followed a control Twitter account that tweeted no content Intervention group—participants followed an intervention Twitter account that posted daily tweets promoting flu vaccination. Campaign engagement (e.g., retweet promotional tweets, construct own tweet containing a hashtag) was incentivized with prize raffle entries.	Vaccination rate of both intervention and control: ~45%	No significant difference between intervention and control groups
Hannings et al. [48]	50,000 students and staff (*n* = unknown)	Pretest–posttest design	A campus-wide flu vaccination promotion campaign across: (1) e-mail; (2) digital or paper flyers; (3) onsite signage directing people to the mobile clinic locations; (4) university health center website and staff recommendations; (5) university publications; (6) local radio; (7) social media (Facebook, Instagram, Twitter); (8) student ambassador communication at campus events	Intervention: 2.3% Previous year: 1.6%	+44%
Lee et al. [45]	50,286 individuals aged 18–65 in a local health plan. This table shows the rate of people aged 18–27 (*n* = unknown)	Randomized controlled trial	3 batches of in-app message were sent to participants, with each batch sent every 2 weeks. Participants were randomized into three groups: Group 1: message encouraging flu vaccination with wellness points reward Group 2: message reminding uptake of flu vaccine without mentioning the reward Group 3: did not receive any in-app messages about flu vaccinations. Messages were arranged to send either (i) in a “timely” manner within two days of the expected prescription pickup by the person at pharmacy in which they may receive flu vaccine, or (ii) on a random delivery date	Intervention groups (messaged): 16% Control group (no message): 13.5%	+18.5% messaged group vs. control No significant difference between group 1 (message + incentive) vs. group 2 (reminder message only)

^1^ Behavioral change was measured by changes in flu vaccination rate against baseline and/or control group. ^2^ Baseline refers to previous year vaccination rate among target audience group(s) of similar characteristics and in the same campus, unless others specified.

**Table 3:** Program assessment against social marketing components.

Study	Behavioral Change ^1^	Audience Segmentation	Consumer Orientation ^2^	Competition	Theory	Insight	Exchange	Marketing Mix
Koharchik et al. [42] ^3^	√ (+7%)	✕	√ Lower level	✕	✕	✕	√ Raffles	√ All 4Ps
Nyandoro et al. [51] ^3^	√ (+54%)	✕	√ Higher level	√ Off-campus vaccine at a fee	✕	√	✕	√ All 4Ps
Saro-Buendía et al. [52] ^3^	√ (+27%)	✕	√ Lower level	✕	✕	✕	√ ID badge sticker	√ All 4Ps
Shropshire et al. [43]	√ (+28%)	✕	✕	✕	✕	✕	✕	√ All 4Ps
Bronchetti et al. [16]	√ (+119%)	✕	✕	√ Off-campus substitution	√ Nudge theory	✕	√ Incentive	√ All 4Ps
Monn [50]	√ (+78%)	✕	✕	✕	✕	✕	✕	√ Price unknown
Huang et al. [46]	√ (+85%)	✕	√ Higher level	✕	√ Peer influence	√	✕	√ All 4Ps
Roberto et al. [44]	✕	✕	✕	✕	√ Extended Parallel Process Model	✕	✕	√ No place or price
Hargrave [47]	√ (+131%)	✕	✕	√ Free off-campus vaccine (insurance)	√ Theory of planned behavior	✕	✕	√ Promotion not mentioned
Osborne et al. [49]	✕	✕	✕	✕	✕	✕	✕	√ No place or price
Hannings et al. [48]	√ (+44%)	✕	✕	✕	✕	✕	✕	√ All 4Ps
Lee et al. [45]	√ (+18.5%)	✕	✕	✕	✕	✕	√ App points	√ All 4Ps

^1^ Behavioral change was measured by changes in flu vaccination rate against baseline and/or control group. ^2^ Level of consumer orientation accessed according to the MATE taxonomy [37]. ^3^ Flu vaccination programs that were targeted at healthcare students.

**Table 4:** Summary of marketing mix, marketing communication channel and message strategy.

Study	Behavioral Change ^1^	Place	Price	Marcom Channel	Message Appeal *“Message” on Promotional Collaterals*
Koharchik et al. [42] ^2^	+7%	Convenient time and location of flu clinic	Free	Education talks, poster, email, raffles	Poster: an appeal to the moral responsibility that healthcare personnel have to their patients to increase immunization
					Email: info on dates, convenient locations of influenza clinics, and raffles
Nyandoro et al. [51] ^2^	+54%	All hospital placement sites	Free	In-class peer promotion, cohort specific social media, posters	Tagline emphases on duty of care, professional responsibility and accountability: *“Get a flu vaccination to protect you &* *your patients”*
Saro-Buendía et al. [52] ^2^	+27%	On-site vaccination in teaching areas	Free	Education talks, posters, leaflets, videos	*“Let’s be more than 30%* *The sticker is for them. Less flu in 2019”*
Shropshire et al. [43]	+28%	Flu clinic	Low-cost	In-class ppt slides, website banner ads, social media	Information on dates and price of influenza vaccination at campus health center
Bronchetti et al. [16]	+119%	Campus health center	Free (insurance plan or parents)	Emails	*“Flu vaccine (get $30!)”*
Monn [50]	+128%	Three vaccination clinics arranged	Free	Posters, Facebook post, College web portal, health center staff	Influenza information, recommendation to receive flu vaccine, time and locations of vaccination clinics
Huang et al. [46]	+85%	University flu clinic + a new clinic in dormitory	Free	Peer health advisors, posters, emails, social media	Tagline appealing to students’ community identity and sense of collective responsibility: *“To protect not only themselves, but also others in their dormitory”*
Roberto et al. [44]	No change	--	--	Ad exposure experiment	Feal appeal messages (threat vs. efficacy)
Hargrave [47]	+113%	Mobile “no-appointment necessary” clinic	Free (partnership with privacy pharmacy)	--	--
Osborne et al. [49]	No change	--	--	Social media (Twitter)	Tweets promoting flu vaccination
Hannings et al. [48]	+44%	Mobile clinic	Free (insurance plan)	e-mail, flyers, signage, website banner, university publications, local radio, social media, student ambassador	Encouraged the audience to get vaccinated using a *“#FluGA”* slogan as a university-wide campaign (but no benefits or cost info were featured)
Lee et al. [45]	+18.5%	Pharmacy in which participants pick up prescriptions	Free	In-app message (earn wellness points in app for flu vaccination)	App message: *“Get a flu shot next time you visit a pharmacy”*
					App message that highlighted the incentive: *“Flu shot = 200 points* *Get a flu shot next time you visit a pharmacy”*

^1^ Behavioral change was measured by changes in flu vaccination rate against baseline and/or control group. ^2^ Flu vaccination programs that were targeted at healthcare students.

**Table 5:** Key learnings in influencing seasonal influenza vaccination behavior.

**What worked**	**Studies**
Free flu vaccine	[16,42,45,46,47,48,50,51,52]
Convenient flu shot locations	[42,45,46,47,48,50,51,52]
Multi-channel communications	[42,43,46,48,50,51,52]
Positive parental and peer influence	[43,46,52]
Incentive	[16,42]
Ads on university website	[43]
Vaccination reminder	[42]
**What did not work**	**Studies**
Social media	[43,49]
Education	[42]
Non-monetary nudges	[16]
Fear-appeal message	[44]
Repeated message	[45]
**What else needs to be addressed**	**Studies**
Lack of time	[42,52]
Intention not to get vaccinated	[16,42]
Lack of follow-through on intentions	[16]

## Data Availability

Not applicable.
